# Extrachromosomal DNA: A New Therapeutic Frontier for Overcoming Cancer Drug Resistance

**DOI:** 10.3390/cells15201834

**Published:** 2026-10-09

**Authors:** Dooil Jeoung

**Affiliations:** Department of Biochemistry, Kangwon National University, Chuncheon 24341, Republic of Korea; jeoungd@kangwon.ac.kr

**Keywords:** extrachromosomal DNA, immune evasion, intratumoral heterogeneity, oncogene amplification, therapy resistance, precision oncology

## Abstract

Extrachromosomal DNAs (ecDNAs) are acentric circular DNA molecules that frequently harbor amplified oncogenes and their regulatory elements. Although ecDNAs occur at low levels in normal cells, oncogenic ecDNAs are enriched in cancer, where they promote tumor evolution, intratumoral heterogeneity, and therapeutic resistance. Advances in long-read sequencing, single-cell multi-omics, and high-resolution imaging have provided new insights into their biogenesis, structure, inheritance, and function. EcDNAs can arise through chromothripsis, breakage–fusion–bridge cycles, replication stress, and aberrant DNA repair, all of which lead to the excision, circularization, and amplification of genomic fragments. Because they lack centromeres and telomeres, ecDNAs undergo dynamic copy-number variation, enhanced chromatin accessibility, enhancer rewiring, and non-Mendelian segregation, thereby facilitating rapid adaptation to selective pressures. EcDNAs contribute to resistance to chemotherapy, targeted therapy, and radiotherapy, and they may also promote immune evasion and immunotherapy resistance through oncogene amplification, enhancer-driven transcription, ecDNA hub formation, and remodeling of the tumor microenvironment. Here, we review recent advances in ecDNA biogenesis, inheritance, and function, with an emphasis on therapeutic resistance, and we discuss emerging strategies that target ecDNA formation, maintenance, and segregation, as well as ecDNA hubs, DNA damage response (DDR) pathways, and replication stress. Collectively, these advances highlight the potential of ecDNA-based biomarkers and ecDNA-directed therapies in precision oncology.

## 1. Introduction

Cancer development is driven by the progressive accumulation of genetic and epigenetic alterations. The etiology of cancer remains largely elusive. Both tumor initiation and tumor progression depend on mechanisms that rapidly modify gene dosage and gene expression, thereby allowing cancer cells to adapt to selective pressures. Cancer is characterized by extensive molecular, cellular, and tissue heterogeneity and undergoes continuous clonal evolution. Over the past several decades, a growing body of evidence has established extrachromosomal amplification as a major driver of tumor initiation, tumor progression, and therapeutic resistance. Extrachromosomal circular DNAs are circular, acentric DNA molecules that are derived from chromosomal DNA and that frequently harbor amplified oncogenes together with their regulatory elements, thereby promoting malignant evolution.

Extrachromosomal circular DNAs comprise a heterogeneous group of circular DNA molecules that are broadly classified into several types: microDNAs (~100–400 bp), larger extrachromosomal circular DNAs that range from hundreds of base pairs to several megabases, telomeric circles (t-circles), extrachromosomal ribosomal DNA circles (ERCs), and large oncogene-containing extrachromosomal DNAs (ecDNAs), which range in size from hundreds of kilobases to several megabases.

The various classes of extrachromosomal circular DNAs perform distinct biological functions. Telomeric circles (t-circles) contribute to alternative lengthening of telomeres (ALT), a telomerase-independent mechanism by which telomeres are maintained in certain cancers [1]. ERCs, in turn, have been implicated in replicative aging in yeast [2]. ERCs have been shown to play important roles in ribosome biogenesis during germ cell development in Xenopus laevis [3]. Collectively, extrachromosomal circular DNAs have been identified in organisms as diverse as yeasts, plants, insects, protozoa, and mammals.

The first cytogenetic evidence for ecDNA emerged in the early 1960s, when double minutes (DMs) were discovered in cancer cells [4]. These extrachromosomal chromatin bodies were smaller than normal chromosomes and lacked centromeres. Although ecDNAs typically lack functional centromeres and telomeres, they remain able to replicate using the normal cell’s DNA replication machinery. EcDNAs have been detected in some normal cells and tissues, but they are far more prevalent in cancer cells, in which they frequently drive high-level oncogene amplification. EcDNAs play critical roles in cancer progression, metastasis, and therapeutic resistance [5].

Subsequent studies demonstrated that DMs harbor amplified oncogenes together with the enhancers, promoters, and other regulatory elements that drive exceptionally high levels of gene expression [6,7,8]. Indeed, oncogenes encoded on ecDNAs rank among the most highly expressed genes in the transcriptomes of many human cancers [9].

Unlike bacterial plasmids, ecDNAs possess no dedicated partitioning system to ensure faithful inheritance. They instead associate transiently with mitotic chromosomes and segregate stochastically into daughter cells during cell division [10]. This unequal distribution generates substantial cell-to-cell variation in ecDNA copy number and so promotes intratumoral heterogeneity [11]. EcDNAs additionally promote tumor growth by mediating oncogene amplification and extensive genomic rearrangements that dynamically remodel the cancer genome [12,13,14].

## 2. Biogenesis of ecDNAs

Genomic instability, one of the hallmarks of cancer, promotes the formation of ecDNAs and thereby facilitates oncogene amplification, tumor evolution, and therapeutic resistance [15]. Several distinct genomic processes can give rise to ecDNAs, among them aberrant DNA damage repair [16], replication stress [17], oxidative stress-induced DNA damage [18], and catastrophic chromosomal rearrangements such as chromothripsis [19,20,21].

Several DNA repair processes have been implicated in ecDNA biogenesis, among them homologous recombination (HR) [22], classical non-homologous end joining (c-NHEJ) [23], and breakage–fusion–bridge (BFB) cycles, which generate the repeated chromosome breakage and amplification events that promote ecDNA formation [24]. Acting together, these pathways facilitate the excision, circularization, and amplification of chromosomal DNA fragments and so lead to the generation of ecDNAs.

Chromothripsis is a catastrophic genome remodeling event in which one or a few chromosomes undergo extensive fragmentation during a single cellular crisis and are then reassembled in an error-prone manner. Micronucleus formation, replication stress, chemotherapy-induced DNA damage, and premature chromosome condensation can all trigger this process. Whereas genomic alterations usually accumulate gradually, chromothripsis generates numerous structural rearrangements in a single event and thereby promotes ecDNA formation [20]. As the shattered chromosomal fragments are aberrantly reassembled, some of them escape linear rejoining and instead circularize into ecDNAs. Such ecDNAs frequently harbor amplified oncogenes, among them *epidermal growth factor receptor* (*EGFR*) [8], *MYC* [25], and *Cyclin D1* [26]. Chromothripsis itself has been identified in a wide range of human cancers, including lung cancer, breast cancer, glioblastoma, melanoma, and colorectal cancer [27,28,29].

HR-mediated DNA repair is essential for maintaining genome integrity during DNA replication. Inhibition of HR can suppress tumor growth and increase the sensitivity of pancreatic ductal adenocarcinoma (PDAC) cells to anticancer agents such as gemcitabine and olaparib [30]. Replication stress, which is characterized by slowed or stalled replication fork progression, activates the DDR, particularly through the ataxia telangiectasia and Rad3-related (ATR)–checkpoint kinase 1 (CHK1) signaling pathway [31]. When persistent, replication stress can compromise genome stability and promote the formation and amplification of ecDNAs, thereby facilitating tumor evolution and resistance to anticancer therapies [32]. Several anticancer agents, including cisplatin, 5-fluorouracil (5-FU), doxorubicin, and camptothecin, induce replication stress by interfering with DNA replication or by generating DNA lesions [33,34,35,36]. In addition, reactive oxygen species (ROS) constitute an important source of replication-associated stress and genome instability. Excessive ROS accumulation can promote genomic instability and therapeutic resistance in leukemia, further supporting a role for oxidative stress in genome remodeling during cancer progression [37].

**Figure 1:**
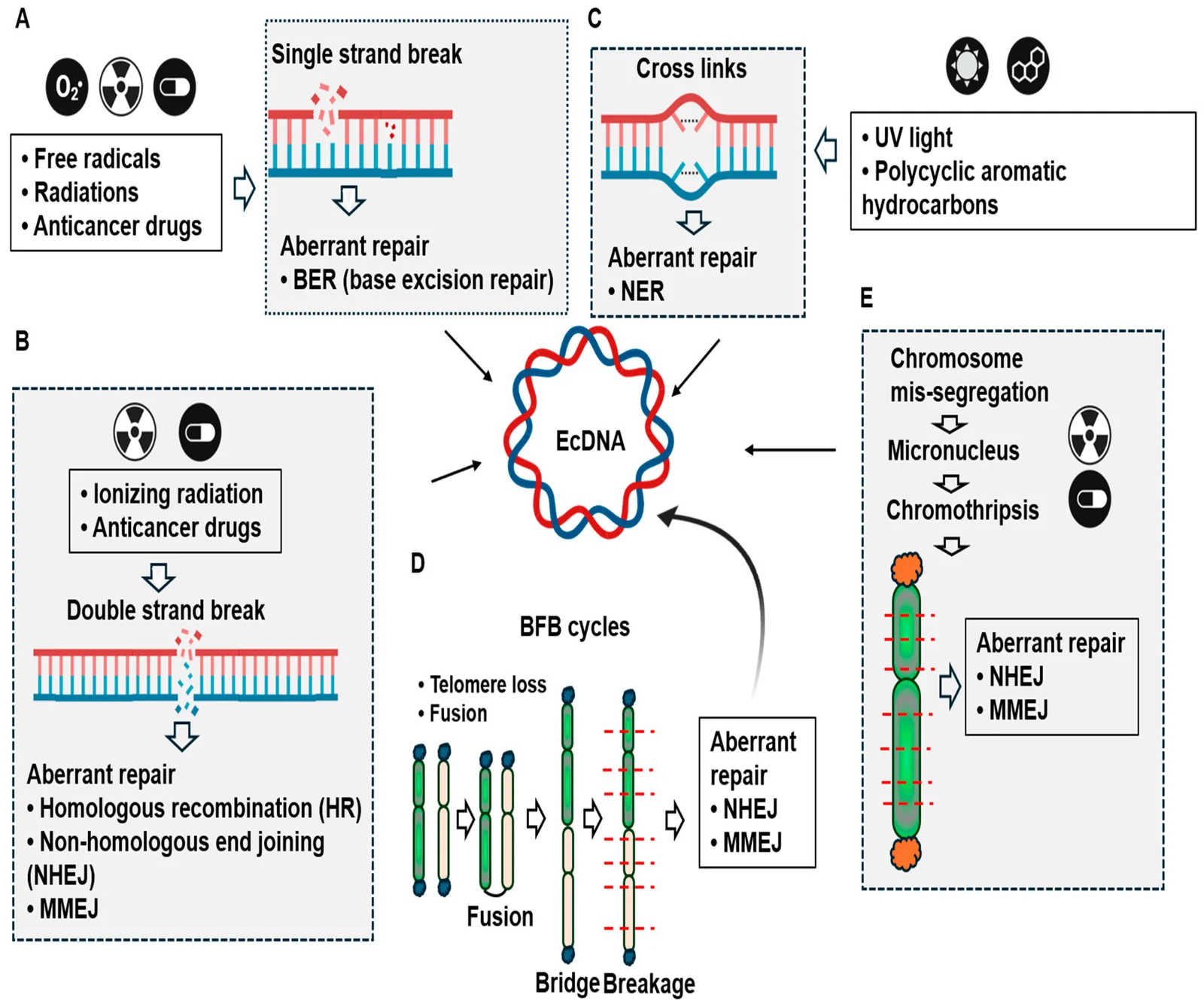
Molecular mechanisms underlying ecDNA biogenesis. (**A**) Single-strand breaks (SSBs) may contribute to ecDNA formation, and base excision repair (BER) together with associated DNA repair defects may facilitate the generation or persistence of the resulting DNA fragments. (**B**) Double-strand breaks (DSBs) can initiate ecDNA formation by generating excised chromosomal DNA fragments that undergo aberrant end joining and circularization. Processing of these fragments through HR, NHEJ, or microhomology-mediated end joining (MMEJ) yields circular extrachromosomal DNA. (**C**) Nucleotide excision repair (NER) is activated by DNA lesions induced by ultraviolet (UV) irradiation and by polycyclic aromatic hydrocarbons (PAHs). Aberrant excision or repair may generate DNA fragments that undergo further processing and circularization and thereby contribute to ecDNA formation. (**D**) Telomere dysfunction or telomere loss can result in chromosome-end fusion and in the formation of dicentric chromosomes. In the subsequent mitosis, these chromosomes form anaphase bridges that can break at sites of mechanical or replication-associated stress. Repeated cycles of chromosome breakage and fusion generate complex rearrangements and amplified DNA fragments, some of which may then undergo aberrant repair and circularization to form ecDNA. (**E**) During mitosis, a lagging chromosome or an acentric chromosome fragment may fail to be incorporated into the primary daughter nucleus and instead become enclosed within a micronucleus. Micronuclei often exhibit defective nuclear envelopes, aberrant DNA replication, and elevated replication stress and DNA damage. Rupture of the micronuclear envelope can subsequently expose the encapsulated chromatin to extensive DNA fragmentation and chromothripsis. The resulting DNA fragments may then undergo error-prone repair and rejoining, generating complex genomic rearrangements and, in some cases, extrachromosomal circular DNA (ecDNA). Figure created using BioRender (biorender.com).

The BFB cycle is a recurrent process that is initiated by telomere loss or dysfunction, which leaves chromosome ends unprotected [38]. Whereas chromothripsis can generate ecDNAs through a single catastrophic chromosome-shattering event, BFB-driven ecDNA formation is thought to occur progressively, through repeated cycles of chromosome fusion and breakage. Once telomeres become dysfunctional, the exposed chromosome ends are recognized as DNA double-strand breaks and serve as substrates for DNA repair, which leads to end-to-end chromosome fusion. In the subsequent mitosis, the resulting dicentric chromosomes are pulled toward opposite spindle poles, so that the chromosome breaks and new, unstable ends are generated. These ends can undergo further rounds of fusion and breakage, thereby establishing recurrent BFB cycles that drive progressive oncogene amplification and genomic instability [38,39]. BFB-associated rearrangements can also generate diverse structural alterations, such as deletions, duplications, inversions, and translocations [24]. Taken together, these findings indicate that genomic instability promotes ecDNA formation, which in turn can confer therapeutic resistance through oncogene amplification. Figure 1 illustrates the molecular mechanisms that underlie ecDNA formation.

## 3. Structural Features of ecDNAs

### 3.1. EcDNAs Display Random Segregation and Extensive Genomic Rearrangements

**Figure 2:**
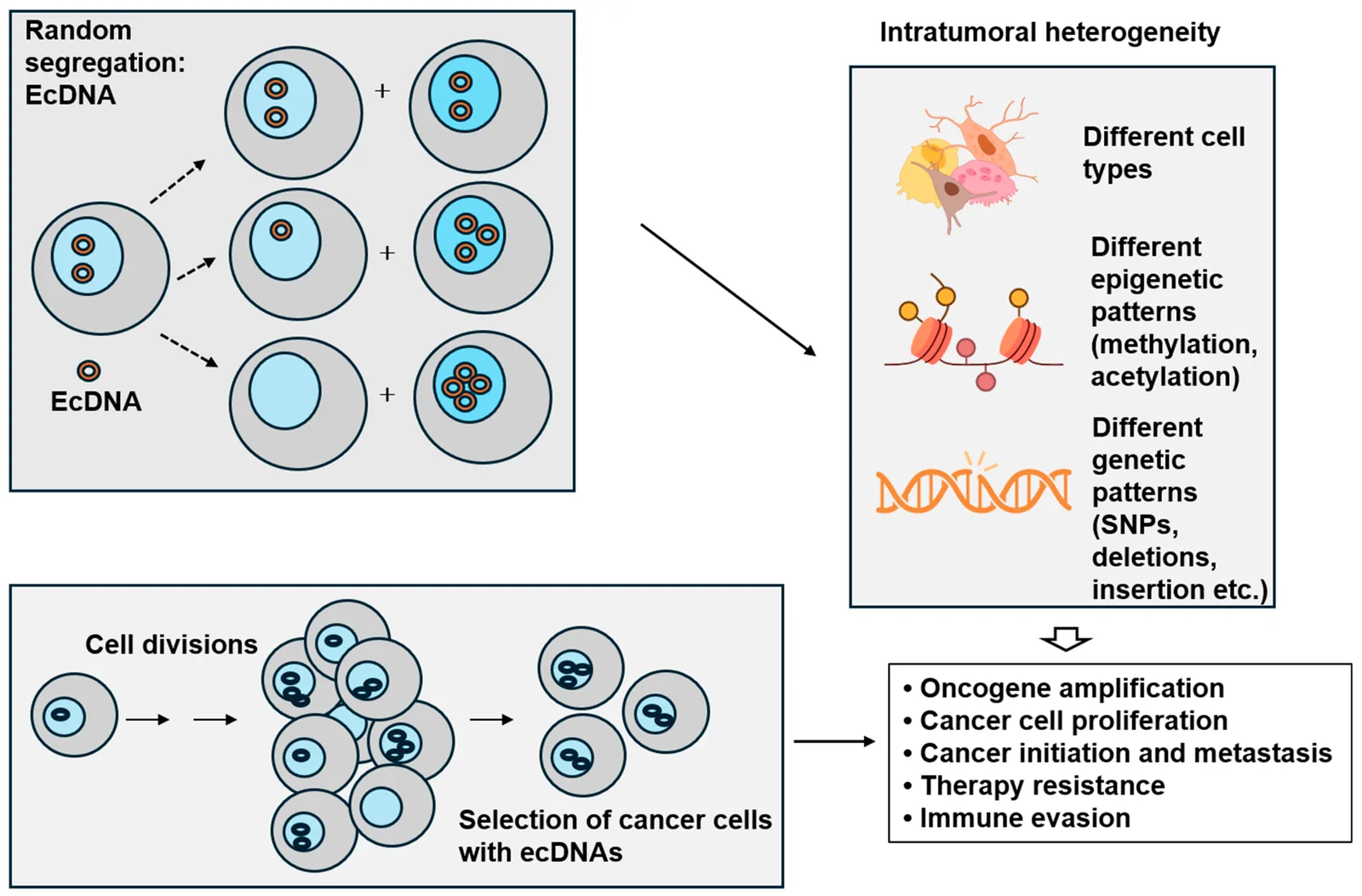
Random segregation of ecDNAs promotes intratumoral heterogeneity. Because ecDNAs lack centromeres and therefore do not undergo canonical kinetochore-mediated chromosome segregation, their inheritance during mitosis is stochastic, so that ecDNA copy number and oncogene dosage vary among daughter cells. This nonuniform segregation contributes to intratumoral genetic and phenotypic heterogeneity and enables rapid adaptation to changing selective pressures. Through dynamic inheritance and clonal selection, tumor cell populations may become enriched for subclones that harbor higher ecDNA copy numbers and show elevated oncogene expression, thereby promoting tumor progression, therapeutic resistance, and immune evasion. Figure created using BioRender (biorender.com).

EcDNAs are circular DNA molecules without free DNA ends, a topology that renders them relatively resistant to exonuclease-mediated degradation. Chromosomal amplifications, by contrast, are maintained through centromere-dependent chromosome segregation during mitosis, so that the amplified genomic regions are inherited relatively stably by daughter cells. Because ecDNAs lack centromeres, they do not undergo conventional chromosome segregation and are instead distributed among daughter cells in a largely stochastic manner; the result is dynamic change in ecDNA copy number, increased intratumoral heterogeneity, and rapid adaptation to therapeutic pressure (Figure 2) [40,41]. Recent studies have identified human genome-derived retention elements that tether ecDNAs to mitotic chromosomes, thereby facilitating their inheritance and contributing to their persistence through cell division [42]. Structurally, ecDNAs differ substantially from linear chromosomal DNA and frequently contain complex genomic rearrangements, such as multiple breakpoint junctions, inversions, duplications, insertions, and deletions. An individual ecDNA can even carry DNA fragments derived from several genomic loci or from different chromosomes, which highlights the complexity of the mechanisms that underlie their formation and subsequent genomic evolution [43].

### 3.2. EcDNAs Exhibit an Open Chromatin Configuration

**Figure 3:**
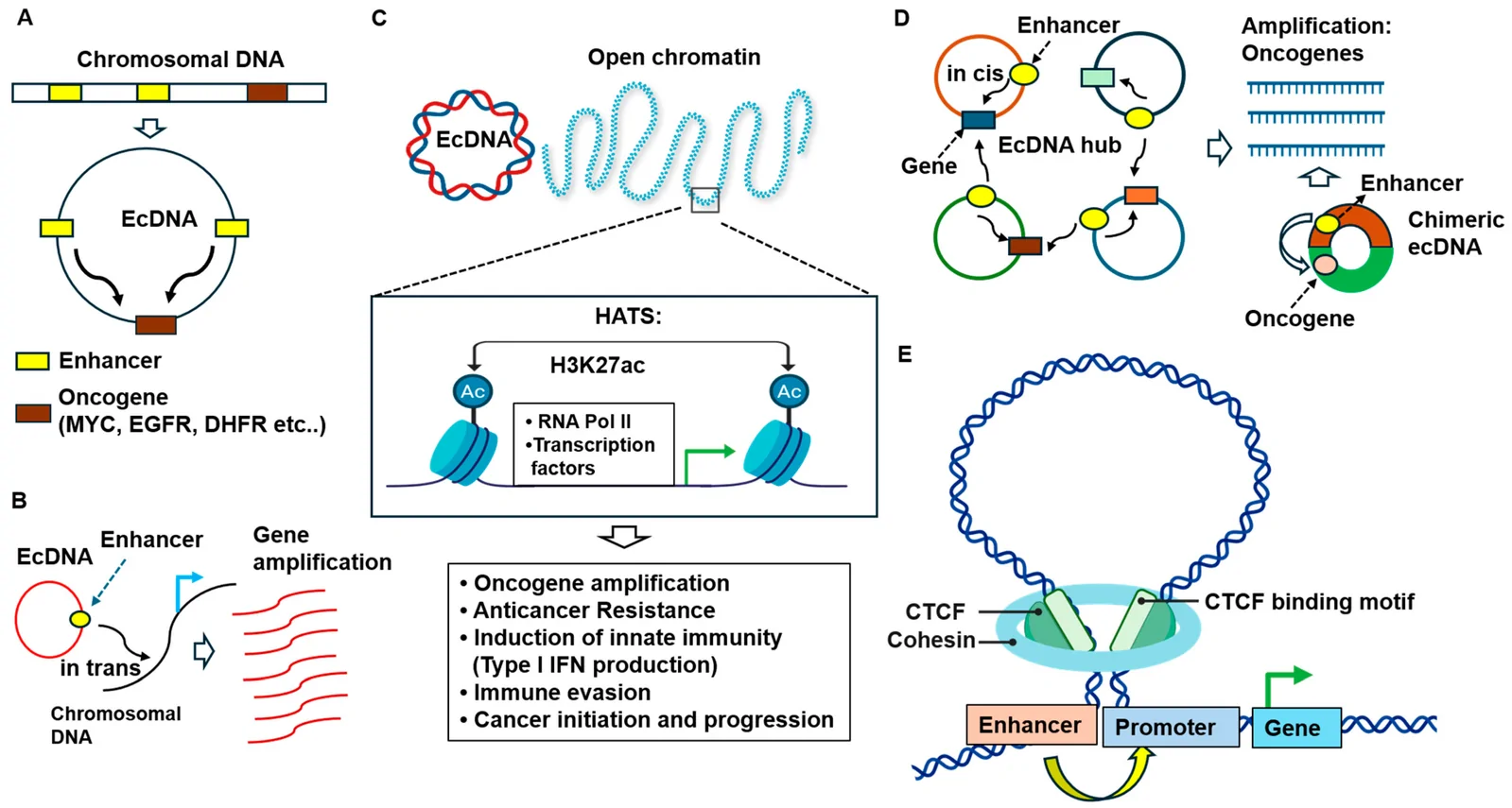
ecDNAs organize open chromatin and form ecDNA hubs. (**A**) ecDNAs arise through the circularization of chromosomal DNA fragments and can contain oncogenes, enhancers, super-enhancers, and other regulatory elements. (**B**) Enhancers located on ecDNAs can also act on chromosomal genes through long-range enhancer–promoter interactions and thus alter gene expression beyond the ecDNA itself. (**C**) Relative to chromosomal DNA, ecDNAs often exhibit highly accessible and transcriptionally active chromatin, which is characterized by altered nucleosome organization, enrichment of active histone modifications such as H3K27ac, and extensive enhancer–promoter interactions in both cis and trans. HATs denote histone acetyltransferases. (**D**) Multiple ecDNA molecules can cluster into transcriptionally active ecDNA hubs that are enriched in transcriptional and chromatin regulators such as MED1, BRD4, and CTCF. These hubs facilitate intermolecular as well as intramolecular enhancer–promoter interactions and thereby promote high-level oncogene transcription. Chimeric ecDNAs contain sequences derived from two or more distinct genomic regions. (**E**) CTCF-mediated chromatin looping can facilitate enhancer–promoter interactions within and between ecDNA molecules and so promote oncogene transcription. Figure created using BioRender (biorender.com).

Enhancers located on an ecDNA can act on oncogene promoters in cis (Figure 3A) or on promoters carried by other DNA molecules in trans (Figure 3B), and in both cases oncogene expression is enhanced. EcDNAs adopt an open, euchromatic configuration that is highly permissive for transcriptional activity (Figure 3C) [44]. Relative to chromosomal DNA, ecDNA chromatin is generally more accessible and is enriched in active histone modifications such as H3 lysine 27 acetylation (H3K27ac), H3 lysine 4 trimethylation (H3K4me3), and H3 lysine 56 acetylation (H3K56ac) [45,46]. During mitosis, ecDNAs can get associated with mitotic chromosomes through interactions with chromatin-associated factors; this facilitates their inheritance and enables transcriptionally active chromatin to be rapidly re-established after cell division [41]. The highly accessible chromatin landscape of ecDNAs also promotes the recruitment of transcription factors and RNA polymerase II and thereby supports robust transcription of the genes they encode [47].

After mitosis, multiple ecDNA molecules can cluster within the nucleus into specialized transcriptional condensates known as ecDNA hubs, in which they colocalize with RNA polymerase II, transcriptional coactivators, and enhancer-associated proteins (Figure 3D) [40,48,49]. These dynamic hubs support robust oncogene transcription and can coordinate transcriptional regulation within ecDNA-rich nuclear compartments [40,48]. They also enable novel enhancer–promoter interactions to form both within an individual ecDNA molecule and between distinct ecDNAs, so that an enhancer carried on one molecule can activate a gene located on another in trans (Figure 3D) [50]. Recruitment of RNA polymerase II, bromodomain-containing protein 4 (BRD4), Mediator complex subunit 1 (MED1), and other enhancer-associated factors further reinforces this cooperative transcriptional environment (Figure 3D) [40,49]. Many ecDNAs harbor amplified oncogenes, among them *MYC* [5], *EGFR* [8], *MDM2* (mouse double minute 2) [51], and DHFR (dihydrofolate reductase) [52]. Chimeric ecDNAs, which contain DNA sequences derived from several genomic regions, can in addition juxtapose oncogenes with regulatory elements and thereby enhance oncogene expression (Figure 3D).

EcDNAs also display remarkable structural plasticity: they acquire or lose DNA segments, recombine with other ecDNA molecules, change dynamically in copy number, reintegrate into chromosomal DNA, and regenerate after anticancer therapy [53,54,55]. Such ongoing structural remodeling generates extensive genomic diversity and thereby facilitates tumor evolution and adaptation to therapeutic pressure [56,57].

## 4. Anticancer Drug Resistance

### 4.1. EcDNAs Can Facilitate Adaptation to Anticancer Therapy

**Figure 4:**
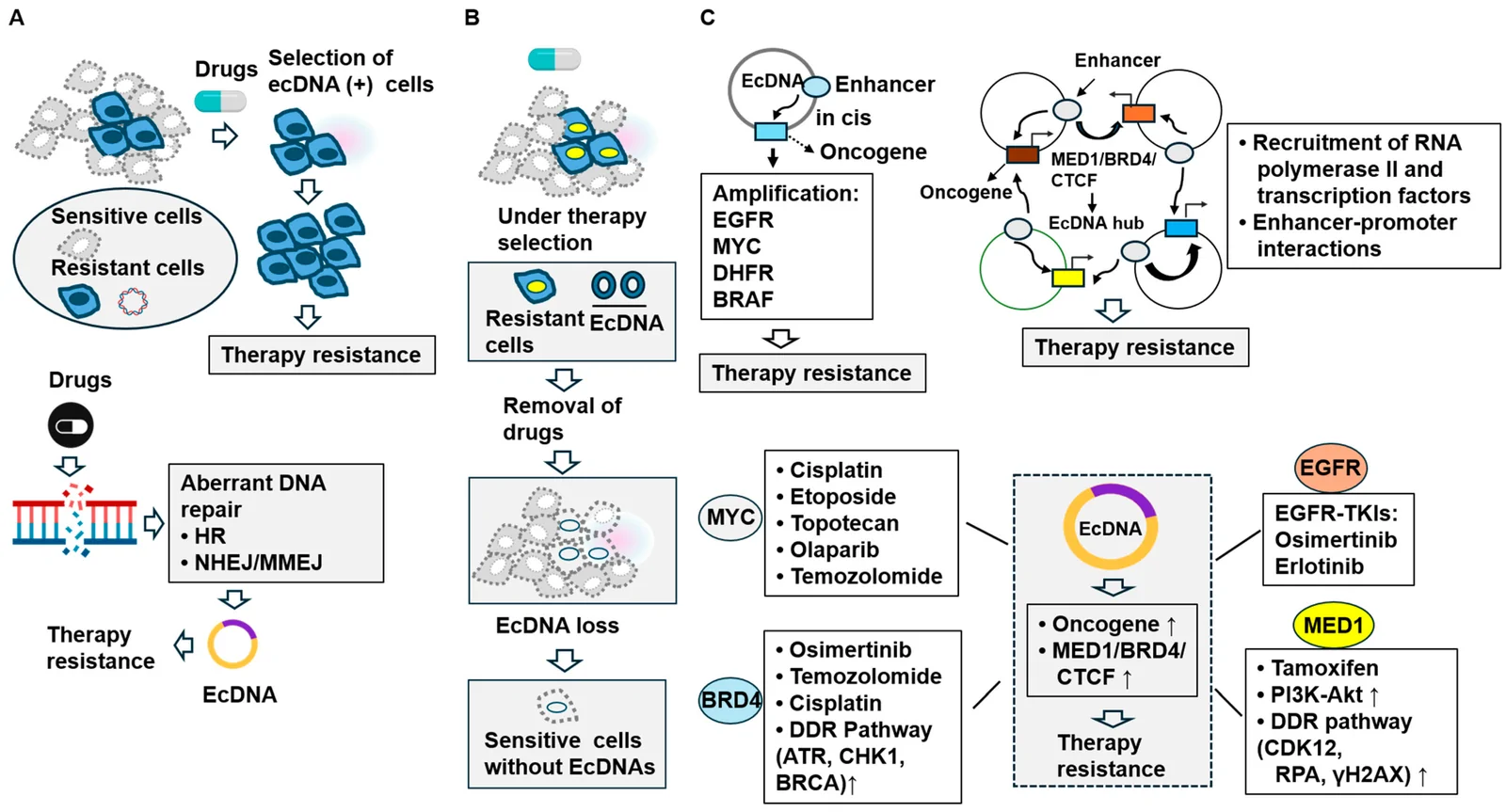
ecDNA promotes therapy resistance. (**A**) Chemotherapy can exert a selective pressure that enriches pre-existing ecDNA-positive cancer cells, which may be more resistant to treatment (**upper** panel). Alternatively, chemotherapy-induced DNA damage may promote aberrant or error-prone DNA repair and ecDNA formation, with oncogene amplification and increased therapeutic resistance as the result (**lower** panel). (**B**) Therapeutic selection can enrich cancer cell populations that harbor ecDNAs, whereas withdrawal of the selective pressure may lead to ecDNA loss or reduction and thereby potentially restore drug sensitivity. (**C**) ecDNAs can promote oncogene overexpression through enhancer–promoter interactions within an individual ecDNA molecule (in cis) and so contribute to therapeutic resistance. Clustering of ecDNAs into ecDNA hubs can further facilitate enhancer–promoter interactions within and between ecDNA molecules (in cis and in trans) and thereby enhance oncogenic transcription. EcDNA-associated transcriptional regulators such as MED1 and BRD4 can support these transcriptional programs and may contribute to therapeutic resistance. Figure created using BioRender (biorender.com).

Selective depletion of ecDNAs by the Werner syndrome ATP-dependent helicase (WRN) exonuclease enhances the therapeutic efficacy of trehalose dimycolate (TDM)-loaded mesoporous silica nanoparticles (MSNs) in B16-F10 melanoma-bearing mice, suggesting a role of ecDNAs in therapeutic resistance [58]. EcDNA-mediated therapeutic resistance may arise through at least two distinct evolutionary processes. First, therapeutic stress may promote the de novo formation or amplification of ecDNA, followed by selection of cells harboring newly generated resistance-conferring ecDNA. Second, ecDNA-positive subclones may pre-exist within a heterogeneous tumor population and undergo positive selection during treatment, leading to their preferential expansion as therapy-sensitive cells are eliminated. Treatment-associated ecDNA formation highlights the potential value of preventing ecDNA generation or targeting the DNA damage response and repair pathways that may facilitate its emergence. Selection of pre-existing ecDNA-positive clones emphasizes the need to identify and eliminate resistant ecDNA-containing subpopulations. Distinguishing these mechanisms experimentally will be important for determining whether ecDNA-mediated resistance primarily reflects selection of pre-existing heterogeneity or adaptive genome evolution during treatment. Conversely, DNA-damaging chemotherapeutic agents, and cisplatin in particular, can promote ecDNA biogenesis by inducing DNA double-strand breaks and aberrant DNA repair, so that ecDNA abundance rises during treatment (Figure 4A) [59,60]. Under therapeutic pressure, ecDNA-positive cancer cells can gain a selective advantage and become more resistant to treatment (Figure 4B), whereas drug withdrawal can reduce or eliminate ecDNAs and thereby restore sensitivity to subsequent treatment (Figure 4B). In medulloblastoma, ecDNAs have been shown to increase the genomic and phenotypic diversity of tumors [61]. Such heterogeneity provides a substrate for Darwinian selection under therapeutic pressure and thereby favors the survival and expansion of drug-resistant subclones [62,63]. Consistent with this, increased intratumoral heterogeneity has been associated with resistance to platinum-based chemotherapy in ovarian cancer [64]. Collectively, these findings support a model in which ecDNAs promote therapeutic resistance by enhancing tumor plasticity, facilitating clonal evolution, and enabling rapid adaptation to anticancer treatment.

### 4.2. EcDNA-Mediated Oncogene Amplification Can Promote Therapeutic Resistance

EcDNAs frequently carry several oncogenes at once, among them *EGFR*, *MET*, *MYC*, and CDK4 [65]; the result is high-level expression of these genes and, in turn, enhanced tumor growth and therapeutic resistance [66,67]. EcDNAs undergo highly dynamic copy-number changes and frequently mediate oncogene amplification [68,69,70], thereby promoting tumor evolution and therapeutic resistance. As shown in Figure 4C, ecDNAs promote oncogene amplification and expression through enhancer–promoter interactions in cis and in trans.

In pancreatic ductal adenocarcinoma (PDAC), ecDNA-mediated *MYC* amplification enables tumor cells to adapt to changing microenvironmental conditions [71]. *MYC* amplification is also frequently observed in triple-negative breast cancer (TNBC), in which chemotherapy can further increase *MYC* expression, suggesting that therapeutic pressure may favor the persistence or expansion of *MYC*-containing ecDNAs [72]. In small-cell lung cancer, ecDNA-mediated *MYC* amplification has been associated with cross-resistance to multiple therapeutic regimens, including cisplatin plus etoposide, olaparib plus temozolomide, and topotecan [73]. Collectively, these findings indicate that ecDNA-mediated oncogene amplification, particularly of *MYC*, represents an important mechanism through which tumors develop therapeutic resistance.

### 4.3. Therapeutic Selection Reshapes the ecDNA Landscape Through Selection and Formation

*EGFR*-containing ecDNAs segregate stochastically during mitosis, so that ecDNA copy number differs among daughter cells and intratumoral heterogeneity increases. This dynamic heterogeneity is now increasingly recognized as a major determinant of resistance to *EGFR* tyrosine kinase inhibitors (TKIs) in *EGFR*-mutant non-small cell lung cancer (NSCLC), because it facilitates the emergence and selection of drug-resistant subclones [74].

Depending on the selective advantage that an individual ecDNA species confers, anticancer treatment can either enrich or deplete ecDNA-containing tumor cells, whereas withdrawal of therapy can rapidly restore ecDNA abundance [57,75]. Under therapeutic pressure, tumor cells that harbor high-copy-number *EGFR* ecDNAs can be preferentially selected, which reinforces *EGFR* amplification and promotes tumor progression in glioblastoma [10]. Consistent with this adaptive process, osimertinib treatment has been reported to induce or enrich *EGFR*-containing ecDNAs in patients with NSCLC [15]. Besides changes in copy number, ecDNAs undergo dynamic structural remodeling that can generate oncogenic variants such as *EGFRvIII*, which carries an in-frame deletion of exons 2–7 [10]. *EGFRvIII* is frequently associated with tumor progression and has also been implicated in resistance to the *EGFR* inhibitor erlotinib [76]. *EGFR*-containing ecDNA may contribute to anticancer drug resistance by altering the expression of genes involved in drug transport and metabolism, potentially reducing intracellular drug accumulation. In glioblastoma, erlotinib treatment reduces the abundance of *EGFRvIII*-containing ecDNAs, whereas drug withdrawal rapidly restores their copy number; this demonstrates how reversible ecDNA-mediated oncogene amplification can be [77]. A similar phenomenon has been observed in gastric cancer, in which the *fibroblast growth factor receptor (FGFR)* inhibitor pemigatinib depletes *FGFR2*-containing ecDNAs, whereas discontinuation of treatment allows the ecDNA-associated *FGFR2* copy number to recover [78]. Collectively, these findings demonstrate that the abundance and the structure of ecDNAs are both highly dynamic and can be continuously reshaped by therapeutic selection. Anticancer therapy may thus not only select pre-existing ecDNA-bearing resistant cells but also alter the abundance and composition of ecDNA populations, thereby facilitating the emergence, expansion, and maintenance of oncogene-amplified tumor cell populations that contribute to therapeutic resistance.

### 4.4. EcDNA-Mediated Oncogene Amplification Confers Resistance to MAPK Inhibitors

Methotrexate (MTX) treatment promotes *dihydrofolate reductase (DHFR)* amplification on ecDNAs, whereas MTX withdrawal reduces the ecDNA-associated *DHFR* copy number and restores drug sensitivity; this illustrates how dynamic and reversible ecDNA-mediated gene amplification can be [79]. Similarly, ecDNA-mediated amplification of *BRAF* confers resistance to mitogen-activated protein kinase (MAPK) pathway inhibitors such as selumetinib and trametinib in melanoma cells [53]. Consistent with these findings, treatment with *BRAF* inhibitors (BRAFi), either alone or combined with mitogen-activated protein kinase kinase (MEK) inhibitors (MEKi), can promote *BRAF* amplification on ecDNAs and enhance resistance to MAPK-targeted therapy both in metastatic melanoma and in patient-derived xenograft (PDX) models [22]. Taken together, these findings demonstrate that ecDNA-mediated amplification of drug-target genes constitutes a dynamic and reversible mechanism of therapeutic resistance.

### 4.5. EcDNA Hubs Promote Oncogene Amplification and Confer Therapeutic Resistance in ecDNA-Positive Cancers

Several chromatin and transcriptional regulators contribute to the formation and transcriptional activity of ecDNA hubs, among them CCCTC-binding factor (CTCF) [7], MED1 [47], and BRD4 [50] (Figure 3D). BRD4 has been implicated in resistance to the *EGFR* inhibitor osimertinib in colorectal cancer cells [80], and it colocalizes with MED1 to support the proliferation of TNBC cells both in vitro and in vivo [81]. Enriched at ecDNA hubs, BRD4 recognizes acetylated histones through its bromodomains and promotes enhancer–promoter interactions, thereby increasing the expression of oncogenes such as *MYC*, *EGFR*, and *Cyclin D1* [82,83]. BRD4 can also interact with the RNA polymerase II-associated factor I (PAF1) and contribute to temozolomide (TMZ) resistance in glioblastoma [84], and it regulates the expression of DNA damage response (DDR) genes such as *ATR*, *CHK1*, and *BRCA1* [85,86]. Inhibition of BRD4 by a ruthenium(II) complex has been reported to enhance the sensitivity of cancer cells to cisplatin [87]. Together, these findings imply that BRD4 may contribute to therapeutic resistance in ecDNA-positive cancers by coordinately regulating oncogenic transcription and DDR pathways. Targeting BRD4 may accordingly enhance the sensitivity of cancer cells to anticancer therapies by disrupting ecDNA-associated transcription and impairing DDR signaling. It will be necessary to develop sensitive methods for detection of ecDNAs in cancers. This is important for determining the importance of ecDNAs in oncogene amplification and therapeutic resistance. It is necessary to examine whether deletion of ecDNAs would affect the expression of BRD4 and therapeutic resistance.

Among the regulators of ecDNA-driven transcription, MED1 has emerged as an important transcriptional coactivator. Because high ecDNA levels are associated with poor clinical outcomes across multiple cancer types [88], such coactivators are of particular clinical interest. MED1 is frequently overexpressed in breast cancer and has been identified as a predictor of poor prognosis in patients receiving anti-estrogen therapy [89]. In breast cancer, MED1 also promotes tamoxifen resistance through activation of the phosphoinositide 3-kinase (PI3K)–AKT signaling pathway [89], which further supports its role in therapeutic resistance. MED1 is enriched at ecDNA hubs and cooperates with oncogenic transcription factors, including *MYC*, E2F, and NF-κB, to promote robust transcription of ecDNA-associated oncogenes [90,91,92]. MED1 also contributes to DDR and genome maintenance by regulating DNA repair pathways such as HR and NHEJ and by promoting the recruitment of DDR factors such as replication protein A (RPA) and phosphorylated histone H2AX (γH2AX) to sites of DNA damage. These functions may provide a mechanistic link between MED1-dependent transcriptional regulation and genome stability in ecDNA-positive cancer cells [93,94]. It nevertheless remains unclear whether MED1 recruitment precedes, coincides with, or follows ecDNA hub assembly, and defining this sequence will provide further insight into ecDNA-mediated transcriptional regulation and may reveal new therapeutic vulnerabilities. Accordingly, disrupting the interactions of MED1 with oncogenic transcription factors or interfering with its recruitment to ecDNA hubs may represent promising strategies for suppressing ecDNA-driven oncogene expression and overcoming therapeutic resistance.

CTCF has emerged as an important regulator of cancer progression and therapeutic response, through its roles in three-dimensional (3D) genome organization, chromatin looping, transcriptional regulation, and DNA damage responses [95,96,97]. By shaping chromatin architecture and enhancer–promoter communication (Figure 3E), it can alter the regulatory landscapes of oncogenes such as *MYC*, *EGFR*, and *MET* and thereby contribute to tumor progression and therapeutic resistance [98]. CTCF–cohesin-mediated chromatin organization has moreover been implicated in TNBC progression and in the cellular response to docetaxel [99]. Further studies may show how CTCF contributes to anticancer drug resistance in ecDNA-positive cancers and whether CTCF-dependent functions can be therapeutically exploited.

## 5. The Role of ecDNA in Immune Evasion

### 5.1. EcDNA-Positive Cancers Can Exhibit Immune Evasion

**Figure 5:**
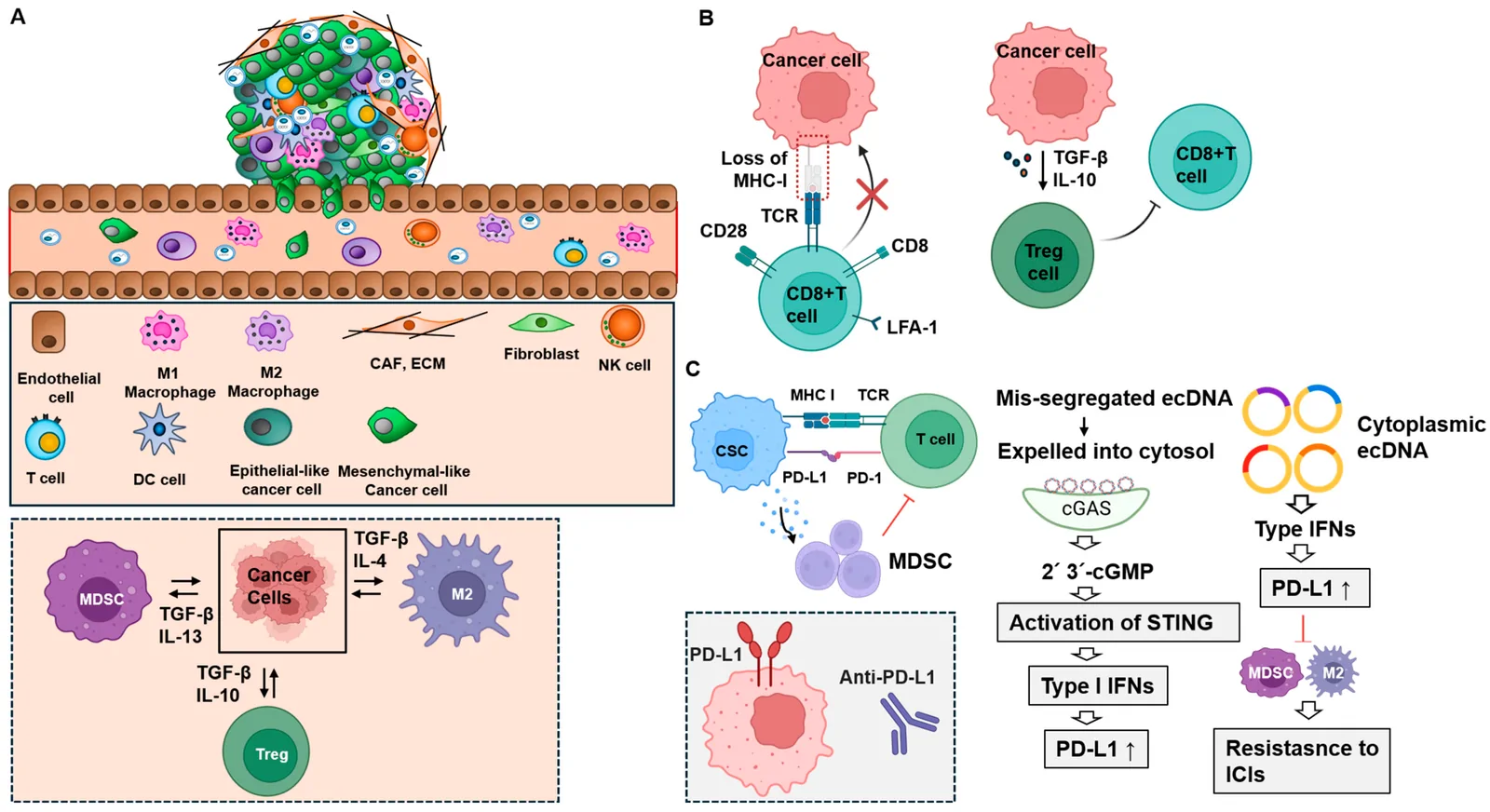
ecDNA-positive cancer cells display immune evasion. (**A**) The tumor microenvironment comprises cancer cells together with a diverse array of immune and non-immune cells (**upper** panel). Cancer cells actively reprogram the immune and stromal cells around them to establish an immunosuppressive microenvironment and thereby promote tumor progression and immune evasion (**lower** panel). CAF, cancer-associated fibroblasts; ECM, extracellular matrix; MDSC, myeloid-derived suppressor cells; M2, M2 macrophages; Treg, regulatory T cells. (**B**) Cancer cells frequently show reduced or lost MHC-I expression, which impairs antigen presentation and limits the cytotoxic activity of CD8^+^ T cells against the tumor (**left** panel). They also promote the recruitment and activation of Treg cells by secreting immunosuppressive cytokines, which suppress the cytotoxic functions of CD8^+^ T cells and so facilitate immune evasion (**right** panel). LFA, lymphocyte function-associated antigen; TCR, T cell receptor. (**C**) Cancer stem cells (CSCs) suppress the cytotoxic activity of CD8^+^ T cells through PD-L1–mediated interactions and promote the recruitment of MDSCs, which inhibit CD8^+^ T-cell function further (**left** panel). Mis-segregated ecDNAs can be expelled into the cytoplasm, where they activate cGAS signaling and induce PD-L1 expression (**middle** panel). The increase in PD-L1 expression may in turn enhance tumor sensitivity to immune checkpoint blockade, including anti-PD-L1 antibodies (**right** panel). Figure created using BioRender (biorender.com).

Therapy-resistant tumors frequently remodel the tumor microenvironment (TME) to suppress antitumor immunity by recruiting regulatory T cells (Tregs), myeloid-derived suppressor cells (MDSCs), and immunosuppressive tumor-associated macrophages (TAMs) (Figure 5A) [100,101,102]. In TNBC, *MYC* overexpression has been associated with an immunosuppressive TME and with increased programmed death ligand 1 (PD-L1) expression, potentially contributing to immune evasion and influencing responses to immune checkpoint inhibitors (ICIs) [103]. Loss or downregulation of major histocompatibility complex class I (MHC-I) expression in cancer cells impairs antigen presentation and CD8^+^ T-cell recognition and thereby facilitates immune evasion (Figure 5B). Cancer cells can also promote the recruitment and activation of Tregs through immunosuppressive cytokines such as transforming growth factor-β (TGF-β) and interleukin-10 (IL-10), which suppress antitumor T-cell responses (Figure 5B). Beyond its function as an immune checkpoint ligand, nuclear PD-L1 interacts with ATR and promotes ATR–CHK1 signaling following genotoxic stress, thereby influencing subsequent cGAS–STING and NF-κB activation [104].

Because ecDNA frequently drives oncogene amplification and extensive transcriptional reprogramming, ecDNA-positive tumors may acquire an immunosuppressive phenotype that facilitates immune evasion and therapeutic resistance. Indeed, ecDNA-containing tumors have been associated with reduced cytotoxic T-cell infiltration and activity, with impaired MHC-I and MHC class II (MHC-II) antigen-presentation programs, and with immunosuppressive TME features [105,106]. Collectively, these findings suggest that ecDNA may contribute to anticancer drug resistance not only through oncogene amplification but also through tumor–immune interactions that promote immune escape.

### 5.2. EcDNAs Can Regulate Responses to Immune Checkpoint Inhibitors

Replication stress, and the chromosomal instability that results from it, can modulate antitumor immunity and influence responses to immunotherapy. In hepatocellular carcinoma, the cyclin-dependent kinase inhibitor p57 (CDKN1C) induces chromosomal instability and enhances CD8^+^ T-cell infiltration, thereby improving the response to anti-programmed cell death protein 1 (PD-1) therapy [105]. The precise contribution of ecDNA remains to be established, but these findings raise the possibility that the genome instability-associated mechanisms that promote ecDNA formation also influence the efficacy of ICIs.

### 5.3. Cytoplasmic ecDNAs Can Enhance Antitumor Immunity by Activating the cGAS–STING Pathway

Cancer-cell PD-L1 engages PD-1 on CD8^+^ T cells and suppresses their activation and cytotoxic function, whereas tumor-derived cytokines and chemokines can promote MDSC recruitment and so reinforce immunosuppression further (Figure 5C). During mitosis, mis-segregated ecDNAs can be excluded from the daughter nuclei and become associated with cytoplasmic DNA structures such as ecDNA-containing micronuclei. These structures can be fragile and undergo membrane rupture, which exposes the ecDNA to cytosolic DNA sensors such as cyclic GMP–AMP synthase (cGAS) (Figure 5C) [106,107,108]. Future studies should determine whether ecDNA depletion modulates cGAS–STING signaling and thereby influences innate immune responses. Genomic instability induced by DNA-damaging stimuli such as ROS and telomere dysfunction can likewise generate cytoplasmic DNA and activate the cGAS–STING pathway [109,110,111]. In multiple myeloma models, PARP1 trapping by talazoparib activates cGAS–STING signaling and promotes immunogenic cell death [112], and cGAS–STING activation can also enhance cytotoxic T-lymphocyte-mediated antitumor immunity in preclinical models [113]. Pharmacological activation of STING has moreover been shown to enhance responses to anti-PD-L1 therapy in mouse tumor models by remodeling the tumor immune microenvironment, in part by reducing immunosuppressive M2-like macrophages and increasing T-cell infiltration [114]. Cytoplasmic DNA generated by genotoxic stress can, in addition, induce PD-L1 expression through cGAS–STING-dependent inflammatory signaling [115]. These findings suggest that cytoplasmic ecDNA may activate the cGAS–STING pathway and induce inflammatory and immune-regulatory programs. However, whether this signaling enhances or limits the efficacy of immune checkpoint inhibitors (ICIs) likely depends on the integrity of downstream immune signaling and the tumor immune microenvironment. Conversely, ecDNA-positive tumors frequently reduce cGAS and STING expression, in part through promoter hypermethylation [116]. Diminished STING expression has also been reported in alternative lengthening of telomeres (ALT)-positive cancers [117]. It is necessary to examine whether ecDNA can directly regulate the expression of c-GAS.

These observations support a model in which cGAS–STING acts as an innate immune barrier to ecDNA-driven tumorigenesis, and in which epigenetic suppression of the pathway allows ecDNA-positive tumors to escape immune surveillance.

### 5.4. EcDNAs Can Promote Immune Evasion Through Oncogene Amplification

EcDNAs that carry immune-modulatory genes can contribute to reduced infiltration of tumor-specific T cells [118]. EcDNA-positive tumors have been reported to exhibit suppression of interferon-γ (IFN-γ)-responsive pathways [119], reduced cytotoxic T-lymphocyte (CTL) activity [120], decreased expression of MHC-I and MHC-II molecules [44], and reduced immune-cell infiltration [25]. Accordingly, this immune-cold phenotype may contribute to the reduced responsiveness of ecDNA-positive tumors to ICIs.

Because impaired antigen-presentation machinery is a characteristic feature of ecDNA-positive tumors, alterations in components of the MHC-I pathway, such as transporter associated with antigen processing 1 and 2 (TAP1/2) and β2-microglobulin (β2M), may also contribute to immune evasion, although this possibility still requires direct experimental validation. *MYC* can in addition promote epithelial–mesenchymal transition (EMT) during cancer progression by activating TGF-β- and WNT-related signaling pathways [121].

EcDNA-driven oncogene amplification may thus promote immune evasion both by transcriptionally reprogramming tumor cells and by remodeling the tumor immune microenvironment. The precise mechanisms involved, including alterations in antigen presentation, innate immune sensing, cytokine signaling, and immune-cell recruitment, nevertheless remain to be fully elucidated.

### 5.5. EcDNA Hubs Promote Immune Evasion Through BRD4 and MED1

EcDNA hubs can drive aberrant transcriptional programs that may alter immune-related gene expression and tumor–immune interactions. Myeloid-cell-specific deletion of BRD4 promotes apoptosis of MDSCs and enhances the efficacy of anti-PD-L1 therapy in lung cancer models [122]. In TNBC, BRD4 promotes PD-L1 expression and contributes to TAM-mediated immunosuppression through induction of granulocyte–macrophage colony-stimulating factor (GM-CSF) [123]. Furthermore, pharmacological or genetic degradation of BRD4 reduces *MYC* and PD-L1 expression, enhances CD8^+^ T-cell infiltration, and attenuates Treg-mediated immunosuppression [124]. Thus, BRD4-dependent transcriptional activity on ecDNA may contribute to immune remodeling and therapeutic resistance, although this mechanism remains to be directly established.

MED1 contributes to the formation of ecDNA hubs, in which it cooperates with enhancer-associated transcriptional machinery to facilitate oncogene amplification [47]. MED1 undergoes DNA damage-dependent SUMOylation following exposure to chemotherapeutic agents such as 5-FU and cisplatin, suggesting that this modification may contribute to cellular responses to chemotherapy-induced DNA damage [125]. Furthermore, truncating mutations in MED1 have been identified in advanced cancers following therapeutic pressure from agents such as tamoxifen and trastuzumab, raising the possibility that MED1 alterations contribute to tumor evolution under therapeutic selection [126]. Together, these findings suggest that MED1 may represent a potential therapeutic target for modulating tumor responses to anticancer therapies, although its clinical relevance requires further investigation.

MED1 may also contribute to tumor immune evasion. In oral squamous cell carcinoma (OSCC), it suppresses CD8^+^ T-cell activity by inducing matrix metalloproteinase 9 (MMP9) and promotes PD-L1 expression through Notch signaling [127]. These findings raise the possibility that inhibiting MED1 could alleviate the immunosuppressive tumor microenvironment and enhance antitumor immune responses.

Thus, ecDNA hub-associated transcriptional amplification may promote immune evasion and therapeutic resistance through sustained BRD4- and MED1-dependent oncogenic and immunosuppressive programs. Accordingly, targeting ecDNA-associated transcriptional dependencies, including these BRD4- and MED1-dependent regulatory mechanisms, represents a promising strategy for overcoming therapeutic resistance and potentially enhancing the efficacy of immune checkpoint blockade.

## 6. Clinical Trials of Molecules Targeting Vulnerabilities Associated with ecDNA or ecDNA Inheritance

### 6.1. Targeting the DDR Represents a Promising Strategy Against ecDNA-Positive Cancers

Therapeutic strategies for ecDNA-positive cancers include direct targeting of ecDNA formation and maintenance, as well as targeting vulnerabilities associated with ecDNA, such as DDR, replication stress, and dependence on DNA repair pathways. DDR pathways are activated in response to DNA damage and can have opposing effects on ecDNA biology. Some DDR mechanisms preserve genome integrity and may therefore suppress ecDNA formation, whereas aberrant or error-prone repair can promote the generation of ecDNA; MMEJ and related repair mechanisms, for example, can contribute to ecDNA biogenesis [128]. Conversely, ecDNA-driven oncogene amplification and the accompanying high transcriptional activity can increase replication stress and DNA damage, potentially creating a dependency on specific DDR pathways for tumor-cell survival. Thus, ecDNA-positive tumors may rely more heavily on DDR pathways to tolerate replication stress and genotoxic stress.

Targeting DDR proteins, including PARP, ATR, DNA-PK, and CHK1, may therefore exploit vulnerabilities associated with ecDNA-driven cancers and, in some contexts, interfere with ecDNA formation or maintenance. DDR proteins also play critical roles in tumor-cell survival following genotoxic therapy. For example, CHK1 has been implicated in resistance to chemotherapeutic agents such as 5-FU and cisplatin in oral squamous cell carcinoma (OSCC) [129]. Accordingly, pharmacological inhibition of DDR pathways represents a promising strategy for enhancing the efficacy of anticancer treatment.

### 6.2. Targeting ATR Can Enhance Sensitivity to Anticancer Therapies

ATR inhibitors such as ceralasertib (AZD6738) and ART0380 increase replication stress by disrupting ATR-dependent replication-stress responses, and they have shown synergistic antitumor activity when combined with chemotherapy, PARP inhibitors, or immune checkpoint blockade in preclinical and clinical studies [130]. ART0380 is currently being evaluated in a Phase I/IIa clinical trial (NCT04657068), as monotherapy and in combination with gemcitabine or irinotecan, in patients with advanced or metastatic solid tumors; the study is designed to assess safety, tolerability, pharmacokinetics, and preliminary antitumor activity [130].

Ceralasertib has also been evaluated in clinical trials, including in lung cancer models (NCT03682289). It induces the expression of galectin-9 (Gal-9), a ligand for the immune checkpoint receptor T-cell immunoglobulin and mucin-domain containing-3 (TIM-3), through STING-type I interferon signaling [131]. Induction of Gal-9 may contribute to adaptive immune resistance during ATR inhibition, whereas blocking Gal-9 can enhance the antitumor activity of ceralasertib, including in combination with anti-PD-1 therapy [131]. Ceralasertib has in addition demonstrated clinical activity in tumors with defects in chromatin regulation. In a Phase II study of patients with ARID1A-deficient solid tumors, it achieved a confirmed objective response rate (ORR) of 14% (4/29 evaluable patients); all four responses occurred in endometrioid endometrial carcinoma or ovarian clear-cell carcinoma, which corresponds to an ORR of 33% in this gynecologic cancer subset [132]. Responding tumors showed increased expression of G2/M checkpoint and DNA double-strand-break-sensing pathways, suggesting that DDR and cell-cycle checkpoint dependencies influence sensitivity to ATR inhibition [132]. Ceralasertib has furthermore been evaluated in combination with the anti-PD-L1 antibody durvalumab in patients with advanced solid tumors, which provides clinical evidence that ATR inhibition can feasibly be combined with immune checkpoint blockade [133].

Collectively, these findings indicate that ATR inhibition exploits replication-stress and DDR vulnerabilities while also modulating antitumor immunity. However, it remains to be determined whether ATR or other DDR inhibitors selectively disrupt ecDNA formation, replication, maintenance, or segregation, or whether they preferentially eliminate ecDNA-containing tumor cells.

### 6.3. Replication Stress at Moderate Levels Contributes to Resistance to Anticancer Therapies

Replication stress represents a double-edged vulnerability in cancer biology, because it contributes to both therapeutic resistance and therapeutic sensitivity. Oncogene amplification, whether of *MYC*, *EGFR*, and other driver genes or through ecDNA-mediated mechanisms, can increase the transcriptional and replicative burden and thereby elevate replication stress [130,134]. At moderate levels, replication stress activates ATR signaling, leading to CHK1 phosphorylation and replication-fork stabilization, which allows cancer cells to tolerate the elevated stress and continue proliferating despite genomic instability. EcDNA-driven oncogene amplification can therefore increase replication stress and potentially activate the ATR–CHK1 pathway, thereby promoting replication-fork protection and contributing to therapeutic resistance [119,135,136]. ATR–CHK1 signaling can also promote cell-cycle checkpoint activation, suppress apoptosis, and enhance tumor-cell survival under genotoxic stress, thereby contributing to resistance to DNA-damaging agents and to targeted therapies such as the PARP inhibitor olaparib in ovarian cancer cells [137,138].

### 6.4. CHK1 Inhibition Exploits ecDNA-Driven Replication Stress to Enhance Sensitivity to Anticancer Therapies

Berzosertib (VX-970), an ATR inhibitor, has shown manageable toxicity and preliminary antitumor activity in patients with advanced or metastatic solid tumors when combined with irinotecan [139]. The CHK1 inhibitor BBI-2779 likewise suppresses gastric cancer progression and enhances sensitivity to the pan-FGFR inhibitor infigratinib [17]. A second CHK1 inhibitor, BBI-355, is currently being evaluated in the Phase I/II POTENTIATE trial (NCT05827614) in patients with advanced solid tumors that harbor oncogene amplifications. Together, these findings suggest that ATR–CHK1 inhibition may be a promising strategy for targeting replication-stress dependencies in oncogene-amplified cancers.

Because ecDNA-driven oncogene amplification can impose substantial replication and transcriptional stress, ecDNA-positive tumors may show increased dependence on ATR–CHK1-mediated stress-response pathways. Pharmacological inhibition of CHK1 may therefore preferentially exploit vulnerabilities in ecDNA-positive and oncogene-amplified tumors by disrupting their adaptation to replication stress. Whether CHK1 inhibition selectively eliminates ecDNA-positive tumor cells or directly disrupts ecDNA maintenance remains to be determined.

### 6.5. Targeting Ribonucleotide Reductase Enhances Sensitivity to Anticancer Therapies

Activation of ribonucleotide reductase (RNR) increases the intracellular availability of deoxyribonucleotide triphosphates (dNTPs) and thereby supports DNA replication and repair, including HR. Increased RNR activity and dNTP supply have been implicated in tumor-cell adaptation to DNA damage and in resistance to anticancer therapies, including PARP inhibitors, platinum-based chemotherapy, and *EGFR* TKIs such as osimertinib [140,141,142].

By increasing transcriptional output and replication demand, ecDNA-driven oncogene amplification can impose substantial replication stress on cancer cells, rendering ecDNA-positive tumors particularly dependent on nucleotide metabolism pathways, including ribonucleotide reductase (RNR) activity, to sustain DNA synthesis and tolerate replication-associated damage. Thus, targeting RNR employes vulnerability of ecDNA-positive cancers.

Beyond its role in nucleotide metabolism and DNA replication, RNR signaling may also contribute to tumor immune evasion. The RNR subunit RRM2 promotes cervical cancer progression and increases PD-L1 expression through DDR-associated signaling, which suggests a link between nucleotide metabolism, genome maintenance, and immune suppression [143]. Conversely, pharmacological inhibition of RNR enhances the antitumor activity of decitabine in lung cancer models, which highlights the therapeutic potential of targeting nucleotide metabolism as part of combination strategies [144,145].

BBI-825, an oral RNR inhibitor, was developed to exploit the increased nucleotide requirements and replication-stress vulnerabilities of oncogene-driven cancers, and it is currently being evaluated in the Phase I/II STARMAP trial (NCT06299761) in patients with advanced solid tumors that harbor resistance-associated gene amplifications. Collectively, these findings suggest that targeting RNR-mediated nucleotide supply may compromise the capacity of genomically unstable and oncogene-amplified tumors to tolerate replication stress and DNA damage. Targeting nucleotide metabolism may accordingly enhance the efficacy of DNA-damaging therapies and help overcome therapeutic resistance.

### 6.6. Targeting ecDNA Segregation Offers a Novel Therapeutic Strategy for ecDNA-Driven Cancers

Disrupting the inheritance of ecDNA during mitosis has emerged as a therapeutic strategy for ecDNA-driven cancers. Disruption of ecDNA partitioning can lower ecDNA copy number and thereby attenuate oncogene dosage and tumor-cell fitness. BBI-940 is an investigational ecDNA-directed therapy (ecDTx) that was designed to exploit vulnerabilities associated with ecDNA maintenance. BBI-940 is an ecDNA-directed strategy that exploits ecDNA-specific/associated dependency. BBI-940 uses a targeted protein degradation (TPD) approach to selectively degrade this kinesin, resulting in ecDNA mis-segregation and reduction of ecDNA in preclinical models.

Recent preclinical studies have demonstrated that pharmacological or genetic degradation of kinesin induces ecDNA mis-segregation and ecDNA depletion and reduces the viability of ecDNA-positive cancer cells, including inducing tumor regression in ecDNA-positive breast cancer models (NCT07408089). BBI-940 therefore represents one of the first therapeutic approaches specifically designed to disrupt ecDNA inheritance rather than to target the amplified oncogene itself. The first-in-human KOMODO-1 trial (NCT07408089) is evaluating BBI-940 in patients with advanced or metastatic breast cancer, including ER+/HER2− disease and TNBC-LAR, both as monotherapy and in combination with fulvestrant. The trial assesses safety, tolerability, pharmacokinetics, and the recommended dose, and ecDNA status will be evaluated retrospectively as a potential biomarker. These studies will help determine whether targeting ecDNA maintenance and segregation mechanisms can selectively impair tumors that depend on ecDNA-mediated oncogene amplification. Further studies are nonetheless needed to identify the kinesins and other mitotic regulators that govern the non-canonical segregation and inheritance of ecDNAs. Table 1 summarizes clinical trials targeting ecDNA segregation and related vulnerabilities, including replication-stress and DDR pathways such as ATR and CHK1.

## 7. Perspectives

Through oncogene amplification, enhancer rewiring, and transcriptional plasticity, it enables cancer cells to adapt to chemotherapy and targeted therapies, and it may also contribute to resistance to immunotherapy. A growing body of evidence indicates that ecDNA additionally promotes immune evasion by altering antigen presentation, tumor–immune interactions, and innate immune signaling. Collectively, these multifaceted functions establish ecDNA as an important driver of therapeutic resistance and identify ecDNA-associated vulnerabilities as promising targets for next-generation anticancer strategies.

Accumulating evidence indicates that anticancer therapy can promote ecDNA formation and alter ecDNA abundance within tumors. It remains unclear, however, whether therapeutic pressure primarily induces the de novo formation of ecDNAs or selectively enriches pre-existing ecDNA-positive subclones, and the two processes may well occur concurrently. The mechanisms behind the rapid and reversible changes in ecDNA copy number during treatment are likewise poorly understood. The molecular basis of ecDNA loss and subsequent re-emergence under therapeutic stress warrants particular investigation, including whether these dynamics reflect de novo ecDNA formation, re-expansion of residual ecDNA, or selection of pre-existing ecDNA-positive cells. Several models of ecDNA biogenesis have been proposed, yet the molecular and cellular determinants that govern when, where, and how ecDNAs arise remain largely unresolved. Future studies should therefore clarify how replication stress, DNA damage, aberrant DNA repair, and oncogene-driven transcriptional and replicative stress interact to promote ecDNA formation. Identifying the genomic regions that are intrinsically susceptible to ecDNA generation, and defining the sequence, chromatin, replication, and DNA repair features that confer this susceptibility, will in addition provide important insights into tumor evolution and therapeutic resistance.

EcDNA-specific therapeutic strategies remain an emerging field, and identifying vulnerabilities that selectively affect ecDNA formation, maintenance, transcription, or inheritance is a critical step toward overcoming ecDNA-driven therapeutic resistance. Current and emerging approaches include the following: (i) inhibiting DDR pathways such as ATR, CHK1, and PARP, so as to exploit the replication-stress and DNA-repair dependencies of ecDNA-driven tumors; (ii) targeting RNR and other nucleotide-metabolism pathways, so as to exploit the increased nucleotide requirements that accompany oncogene amplification and replication stress; (iii) targeting ecDNA-associated transcriptional dependencies by disrupting the formation or function of ecDNA hubs through regulators such as MED1 and BRD4; (iv) interfering with ecDNA-associated chromatin organization and transcriptional programs, including dependencies on RNA polymerase II and on oncogenic transcription factors; (v) targeting the immune-evasion mechanisms of ecDNA-positive tumors through immune checkpoint blockade and other immunotherapeutic approaches; and (vi) directly disrupting ecDNA maintenance and inheritance, including interference with ecDNA segregation during mitosis, as exemplified by the investigational ecDTx BBI-940, as well as experimental approaches such as CRISPR-based ecDNA degradation.

Beyond these approaches, an important area of future research will be to determine whether endogenous cellular pathways can selectively recognize and eliminate ecDNAs. Potential mechanisms may include nucleases involved in DNA quality control, DNA repair-associated nucleases, and cellular pathways that recognize and remove aberrant or damaged DNA structures. Whether autophagy-related or other DNA quality-control mechanisms contribute to ecDNA clearance likewise remains to be determined. Elucidating such pathways could reveal novel therapeutic strategies for selectively eliminating ecDNAs while minimizing toxicity to normal cells. A major challenge in developing ecDNA-directed therapies is achieving sufficient selectivity. Because ecDNAs are generally derived from chromosomal genomic regions, they often retain substantial sequence identity with their chromosomal loci of origin. Therefore, sequence-based targeting alone may not be sufficient to distinguish ecDNA from chromosomal DNA. Therapeutic strategies will instead need to exploit ecDNA-specific features, such as their circular topology, high copy number, abnormal replication or segregation, and distinctive chromatin and transcriptional states, while avoiding damage to essential chromosomal loci and to normal cells. Overcoming this challenge will be essential for translating ecDNA-directed strategies into safe and effective clinical therapies.

Because ecDNA promotes intratumoral heterogeneity, transcriptional plasticity, and rapid adaptation to therapeutic pressure, monotherapy directed at a single ecDNA-associated vulnerability may be insufficient to achieve durable tumor control. Combination strategies that simultaneously target ecDNA-associated dependencies and complementary oncogenic or survival pathways may therefore be required to prevent or delay therapeutic resistance. Potential combinations include ecDNA-directed agents together with inhibitors of DDR pathways, such as ATR, CHK1, or PARP inhibitors, as well as with ICIs, conventional chemotherapy, or other molecularly targeted therapies. Such approaches may suppress the emergence of resistant subclones, exploit multiple vulnerabilities simultaneously, and enhance the durability of antitumor responses. However, the optimal drug combinations, treatment sequences, dosing schedules, and patient-selection biomarkers remain to be determined in clinical studies.

There is growing interest in translating ecDNA research into clinical practice through the development of ecDNA-based biomarkers, prognostic indicators, and ecDNA-directed therapeutic strategies. Although the clinical development of ecDNA-directed therapies remains at an early stage, the first-in-human KOMODO-1 trial (NCT07408089) is evaluating BBI-940, an investigational ecDTx designed to disrupt ecDNA segregation and inheritance. In parallel, other clinical studies seek to exploit vulnerabilities that may be associated with ecDNA-positive tumors, including replication stress, DDR dependencies, nucleotide metabolism, and oncogene amplification. Clinical translation of these approaches nevertheless remains challenging because of the lack of standardized, sensitive, and clinically scalable methods for ecDNA detection and quantification, an incomplete understanding of interpatient and intratumoral heterogeneity in ecDNA structure and composition, and the limited incorporation of ecDNA status into patient stratification.

**Figure 6:**
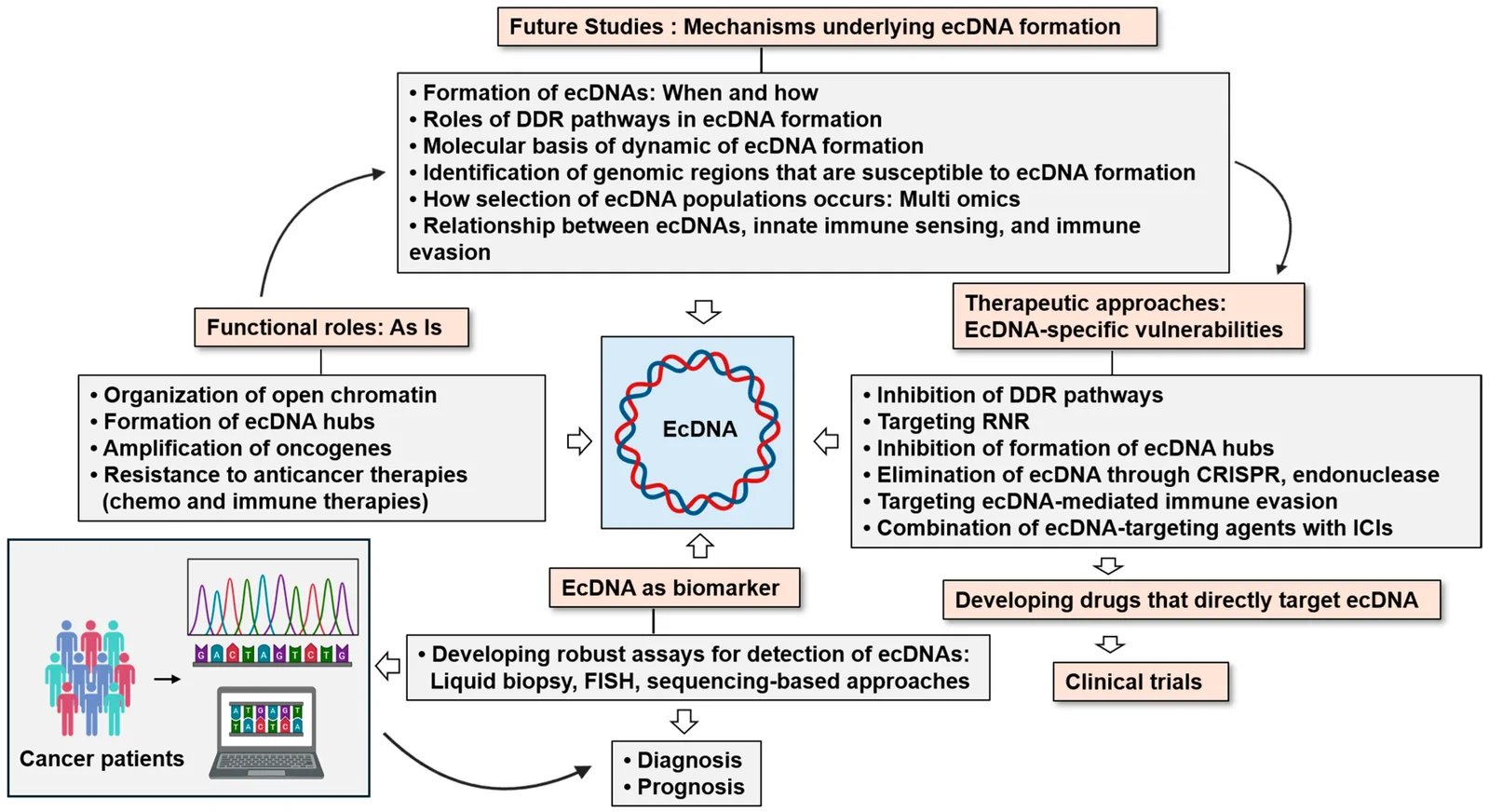
Perspectives on ecDNA research. A comprehensive understanding of the mechanisms that govern ecDNA formation, maintenance, segregation, and transcription is essential if ecDNA biology is to be translated into therapeutic opportunities. Emerging therapeutic strategies increasingly aim to exploit vulnerabilities that are specific to, or preferentially associated with, ecDNA-positive cancer cells. They include targeting the DDR and replication stress pathways so as to inhibit ecDNA biogenesis; disrupting ecDNA maintenance and the asymmetric or non-Mendelian segregation of ecDNA during cell division; and suppressing ecDNA-driven transcription by targeting ecDNA hubs, chromatin regulators, and enhancer activity. Further strategies seek to eliminate ecDNA-containing cancer cells selectively or to exploit their increased dependence on the pathways that preserve genomic stability. Collectively, these approaches may suppress oncogene amplification, constrain tumor evolution and therapeutic resistance, and ultimately improve the efficacy of precision cancer therapies. Figure created using BioRender (biorender.com).

EcDNA can currently be detected and characterized using complementary approaches, including whole-genome sequencing coupled with computational amplicon reconstruction, fluorescence in situ hybridization (FISH), optical genome mapping, and long-read sequencing. WGS-based tools such as AmpliconArchitect and AmpliconClassifier can infer ecDNA structures from complex amplification and rearrangement patterns, whereas FISH enables the direct visualization of ecDNA at the single-cell level. Single-cell sequencing provides information on the distribution and heterogeneity of ecDNA across tumor cell populations. Advances in artificial intelligence (AI), computational genomics, and multimodal data integration may accelerate the field further by improving how ecDNAs are detected and characterized, by identifying ecDNA-associated molecular features, and by predicting therapeutic vulnerabilities. Such technologies could facilitate biomarker-driven patient selection, guide precision treatment strategies, and support the design of ecDNA-directed clinical trials. Figure 6 summarizes the key areas of future research that will be required to establish ecDNA as a clinically useful biomarker and therapeutic target.

## Figures and Tables

**Table 1:** Clinical development of therapeutics targeting ecDNA-specific vulnerabilities.

Title	Phase	Condition or Disease	Prospective Outcome Measures	Dates	ID/Purpose
**A Phase I/IIa, Open-label, Multi-center Study to Assess the Safety, Tolerability, Pharmacokinetics and Preliminary Efficacy of the ATR Kinase Inhibitor ART0380 Administered Orally as Monotherapy and in Combination to Patients With Advanced or Metastatic Solid Tumors**	I/IIa	Advanced or Metastatic Solid Tumors	Drug: ART0380 Drug: Gemcitabine Drug: Irinotecan Primary (original): Maximum tolerated dose (MTD) by the number of participants with dose-limiting toxicities (DLTs) from ART0380 monotherapy and in combination with gemcitabine Number of participants with adverse events following administration of ART0380 Progression-free survival by RECIST 1.1 in participants receiving ART0380 in combination with gemcitabine or gemcitabine alone Secondary (current): - Pharmacokinetic analysis (single and multiples) - Determination of plasma concentration of ART0380 when given alone and in combination - Determination of renal clearance ART0380	27 January 2021 (Start) December 2027 (Completion)	NCT04657068/ Interventional
Phase II Trial of Ceralasertib (AZD6738) Alone and in Combination With Olaparib or Durvalumab in Patients With Selected Solid Tumor Malignancies	II	renal cell carcinoma (RCC), urothelial carcinoma, all pancreatic cancers, endometrial cancer, and other solid tumors that have spread to nearby tissue or lymph nodes.	To assess objective response rate (ORR) of ceralasertib monotherapy and ceralasertib + olaparib by Response Evaluation Criteria in Solid Tumors (RECIST) version 1.1 criteria (ARID1A Cohort) To assess objective response rate of ceralasertib monotherapy in patients with other advanced solid tumor malignancies harboring pathogenic ATM mutations and/or loss of ATM expression by IHC (ATM Cohort). To assess the objective response rate (ORR) of ceralasertib in combination with durvalumab by RECIST 1.1 criteria (Endometrial Cohort)	17 January 2019 (Start) 31 March 2028 (Completion)	NCT03682289/Interventional
An Open-Label, Multicenter, First-in-Human, Dose-Escalation and Dose-Expansion, Phase 1/2 Study of BBI-355 and BBI-355 in Combination With Select Targeted Therapies in Subjects With Locally Advanced or Metastatic Solid Tumors With Oncogene Amplifications	I/II	Locally Advanced or Metastatic Solid Tumors With Oncogene Amplifications	Frequency and severity of treatment-emergent adverse events (TEAEs) of BBI-355 Maximum Tolerated Dose (MTD) and/or Recommended Phase 2 Dose (RP2D) of BBI-355 Maximum observed plasma concentration (Cmax) of BBI-355, erlotinib, futibatinib, and BBI-825 Anti-tumor activity of BBI-355 as a single agent and in combination with erlotinib, futibatinib, or BBI-825 Maximum observed plasma concentration (Cmax) of BBI-355	24 March 2023 (Start) 17 March 2026 (Completion)	NCT05827614/Interventional
An Open-Label, Multicenter, First-in-Human, Phase 1 Study of BBI-940 in Advanced or Metastatic Breast Cancer: Kinesin Oral Molecular Degrader for Oncology (KOMODO-1)	I	Breast cancer and metastatic breast cancer	Rate of dose-limiting toxicities (DLTs) in each BBI-940 monotherapy dose escalation cohort Incidence of treatment-emergent adverse events (TEAEs) in each dose group and overall Incidence of study treatment discontinuation and/or interruption by dose group and overall Objective response rate (ORR) by dose group and overall Progression-free survival (PFS) by dose group and overall Maximum observed plasma concentration (Cmax) of BBI-940	25 February 2026 (Start) 31 May 2029 (Completion)	NCT07408089/Interventional
An Open-Label, Multicenter, First-in-Human, Dose-Escalation and Dose-Expansion, Phase 1/2 Study of BBI-825 and BBI-825 in Combination With Select Targeted Therapies in Subjects With Locally Advanced or Metastatic Solid Tumors With Resistance Gene Amplifications	I	Solid tumor	Frequency and severity of treatment-emergent adverse events (TEAEs) of BBI-825 Maximum Tolerated Dose (MTD) and/or Recommended Phase 2 Dose (RP2D) of BBI-825 Maximum observed plasma concentration (Cmax) of BBI-825 Anti-tumor activity of BBI-825 as determined by RECISTv1.1: Number of participants achieving a best response of progressive disease, stable disease, partial response, or complete response.	28 March 2024 (Start) 25 June 2025 (Completion)	NCT06299761/Interventional
Phase I Clinical Trial of M6620 (VX-970, Berzosertib) in Combination With the Topoisomerase I Inhibitor Irinotecan in Patients With Advanced Solid Tumors	I	Advanced solid tumors	To determine the maximum tolerated dose (MTD) and recommended phase 2 dose (RP2D) of M6620 (VX-970, berzosertib) in combination with irinotecan hydrochloride (irinotecan) in patients with advanced solid tumors. To estimate the safety and tolerability of M6620 (VX-970, berzosertib) in combination with irinotecan. To document anti-tumor activity. III. To determine the pharmacokinetic (PK) and pharmacodynamic (PD) parameters of M6620 (VX-970, berzosertib) and irinotecan.	22 July 2016 (Start) 6 March 2027 (Completion)	NCT02595931/Interventional

## Data Availability

No new data were created or analyzed in this study. Data sharing is not applicable to this article.
